# High-Throughput Sequencing of RNA Silencing-Associated Small RNAs in Olive (*Olea europaea* L.)

**DOI:** 10.1371/journal.pone.0027916

**Published:** 2011-11-28

**Authors:** Livia Donaire, Laia Pedrola, Raúl de la Rosa, César Llave

**Affiliations:** 1 Department of Environmental Biology, Centro de Investigaciones Biológicas, The Spanish National Research Council, Madrid, Spain; 2 Lifesequencing S.L., Parc Científic Universitat de Valéncia, Valencia, Spain; 3 Centro Alameda del Obispo, IFAPA, Córdoba, Spain; United States Department of Agriculture- Agricultural Research Service, United States of America

## Abstract

Small RNAs (sRNAs) of 20 to 25 nucleotides (nt) in length maintain genome integrity and control gene expression in a multitude of developmental and physiological processes. Despite RNA silencing has been primarily studied in model plants, the advent of high-throughput sequencing technologies has enabled profiling of the sRNA component of more than 40 plant species. Here, we used deep sequencing and molecular methods to report the first inventory of sRNAs in olive (*Olea europaea* L.). sRNA libraries prepared from juvenile and adult shoots revealed that the 24-nt class dominates the sRNA transcriptome and atypically accumulates to levels never seen in other plant species, suggesting an active role of heterochromatin silencing in the maintenance and integrity of its large genome. A total of 18 known miRNA families were identified in the libraries. Also, 5 other sRNAs derived from potential hairpin-like precursors remain as plausible miRNA candidates. RNA blots confirmed miRNA expression and suggested tissue- and/or developmental-specific expression patterns. Target mRNAs of conserved miRNAs were computationally predicted among the olive cDNA collection and experimentally validated through endonucleolytic cleavage assays. Finally, we use expression data to uncover genetic components of the miR156, miR172 and miR390/*TAS3*-derived trans-acting small interfering RNA (tasiRNA) regulatory nodes, suggesting that these interactive networks controlling developmental transitions are fully operational in olive.

## Introduction

In eukaryotes, small RNAs (sRNAs) exert transcriptional and post transcriptional control of genome expression to modulate pivotal events in development and response to environmental cues [1]–[3]. Generally speaking, sRNAs are inhibitors of gene expression that act as specificity factors that guide bound effector proteins to target nucleic acids via base-pairing interactions [4]. In the model plant *Arabidopsis thaliana*, sRNA biogenesis is catalyzed by four homologues of the ribonuclease Dicer-like, DCL, that use RNA with double-stranded (ds) features as a substrate [5]. In plants, sRNAs can be broadly classified as microRNAs (miRNAs) and small interfering RNAs (siRNAs) [6], [7]. *MIRNA* genes are transcribed by RNA polymerase II into primary transcripts containing a local stem-loop structure that provides the substrate for DCL1 cleavage into mature miRNAs of 21–22 nucleotides (nts) [8], [9]. miRNAs have a big impact on shaping transcriptomes and proteomes in plants as they negatively target cognate mRNAs for destruction or translational arrest [10]–[12]. Vascular plants including angiosperms (eudicots and monocots), gymnosperms and pteridophytes contain a repertory of ancient miRNAs that are evolutionary conserved and control a large set of fundamental processes in cell homeostasis and function [5], [13]–[15]. Next-generation deep sequencing technologies and computational prediction methods have contributed to markedly expanding our knowledge of the sRNA universe in the eukaryotic cell by bringing into scene a number of newly evolved and species-specific miRNAs [13], [16]–[18]. These non-conserved, “young” miRNAs, that are poorly expressed normally from single genes, provide a means to understand how plant species face the new threats associated to environments that demand specific developmental conditions and stress responses [19]–[21].

At a broad level, the various classes of plant 21- to 24-nt siRNAs derive from longer dsRNA precursors that are processed by DCL2, DCL3 and DCL4 [22]. The biosynthesis of these long dsRNA precursors usually entails the activity of one of several RNA-dependent RNA polymerases (RDRs) that copy single stranded RNA [23]–[25]. Genomic sources of siRNAs include repetitive sequences, transposons, centromeres, convergent mRNA transcripts and other natural sense-antisense pairs, duplexes involving pseudogene-derived antisense transcripts and the sense mRNA from their cognate genes, hairpin RNAs as well as trans-acting siRNA (tasiRNA)-generating transcripts (TAS) [1], [6], [26]–[28]. Endogenous siRNAs inactivate homologous sequences by a variety of mechanisms that include canonical post-transcriptional gene silencing as well as chromatin-dependent gene silencing [29], [30].

Olive (*Olea europaea* L.) is one of the most economically important evergreen fruit crops in the Mediterranean basin. Wild and cultivated olives are diploid (2n = 46) and have a genome size of approximately 1,800 MB [31]. Virgin olive oil, the fruit juice of this species, is worldwide appreciated due to its potential health and nutritional benefits and to its exceptional organoleptic properties. Olive cultivars colonize different environments, characterized by semi-arid climatic conditions at different altitudes, vegetative communities and soils, including those with extreme levels of drought, low temperatures and salinity [32]. From an ecological point of view, olive populations protect soils against desertification due to their great resistance to wind and drought, their ability to re-sprout after fire or frost, and their very long lifespan [32].

The length of the juvenile period in olive trees, longer than other fruit tree species, has been traditionally one of the main drawbacks of fruit tree breeding. Seedlings usually reach flowering from 4 to 7 years after seed germination in intensive growing conditions [33]. Therefore an early selection criteria for short juvenile period is an economical issue since the length of the juvenile period directly correlates with the length of the unproductive period (time between the plantation of a rooted shoot and the first commercial crop) in adult shoots [34]. Traditionally, the occurrence of flowering has been used as a marker of phase change, although a marker based on leaf anatomy has been recently proposed [35]. Little is known however about the set of genes and regulatory circuits controlling juvenile-to-adult transition in olive trees.

Whereas miRNAs have been extensively profiled in a wide variety of herbaceous plant species (http://www.mirbase.org/), the list of miRNAs from wooden plants is scarce and restricted to conifers (*Taxus chinensis*, *Picea abies and Pinus spp.*), poplar (*Populus spp.*), grapevine (*Vitis vinifera*) and citrus (*Citrus spp.*) [18]–[20], [36]–[41]. For olive, a study of sRNAs is lacking. In this paper, we report the first catalogue of sRNAs from olive trees by taking advantages of high throughput pyrosequencing and RNA hybridization technologies. To infer the potential role of miRNAs during growth and development in this plant species, different tissues and developmental stages from different genetic backgrounds (olive varieties) were interrogated. A total of 18 previously known miRNAs were identified within our sequenced set based on sequence homology. Developmental stage- and tissue-specific expression patterns, target identification and target cleavage abilities for selected conserved miRNAs are presented. Finally, we show that the regulatory networks involving miR156 and miR172 nodes as well as tasiRNA-mediated regulation of *Auxin Response Factor* (*ARF*) genes in the control of developmental timing were conserved in olive. This study is part of a collaborative research project named OLEAGEN funded by Genoma España (Spain) which aims to provide genomic tools in olive through the identification of key genes and regulatory pathways involved in quality and production traits, such as fruit and oil composition, length of the juvenile period, and plant architecture.

## Results

### Construction of sRNA libraries in *O. europaea*

**Figure 1:**
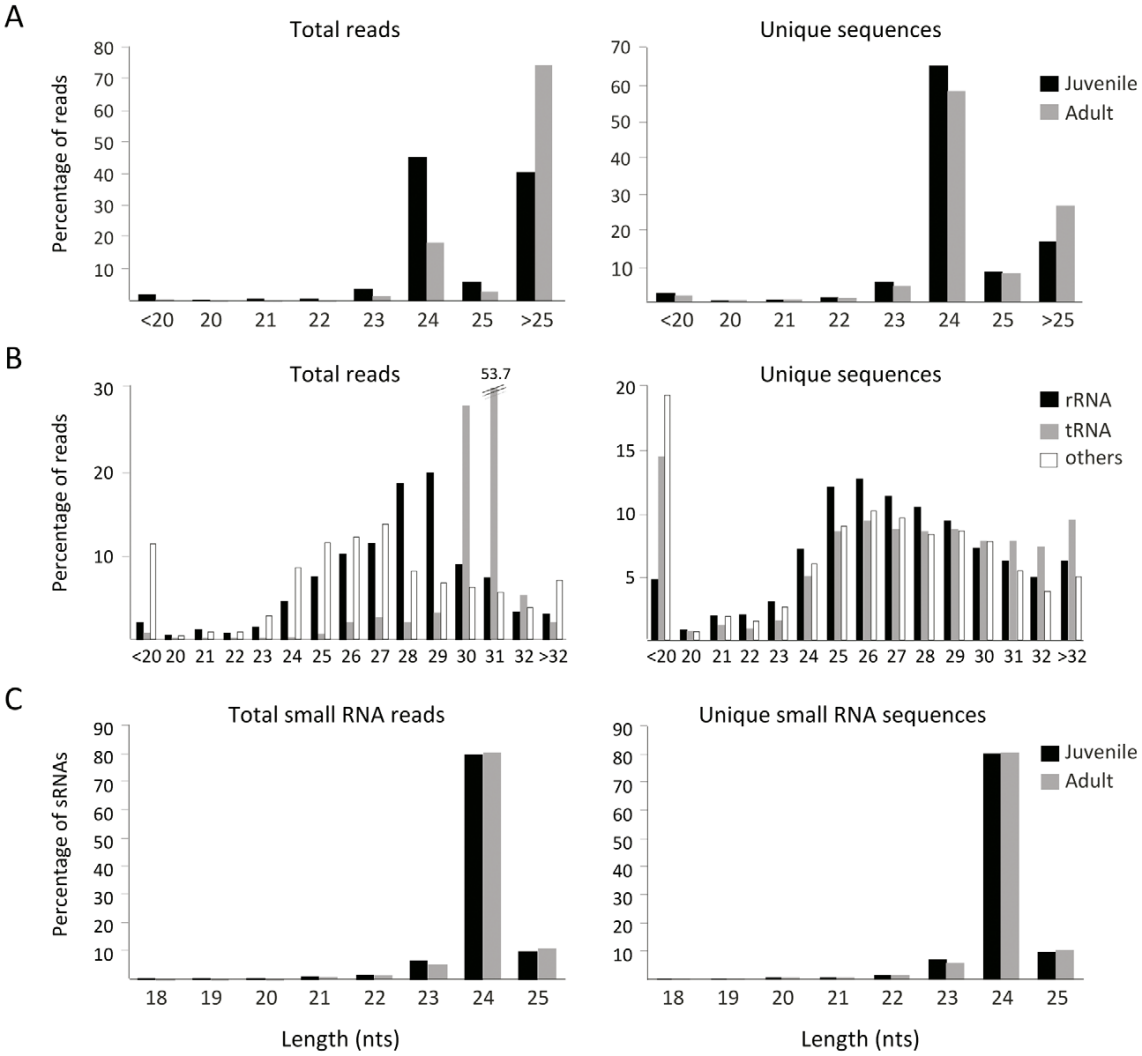
Size distribution of sequenced sRNAs from *O. europaea*. Size distribution of unfiltered (*A*), noncoding RNA-matching (*B*), and high-quality filtered sequences (*C*) from sRNA libraries obtained from juvenile and adult shoots in *O. europaea*. Histograms represent the percentage of total or unique reads/sRNAs within each length class.

High throughput sequencing offers a powerful means for quantitative and qualitative profiling of sRNA populations and it is convenient for exploring sRNAs in plant species such as *O. europaea* from which limited genome information is accessible. In this study, two separate sRNA cDNA libraries were generated from juvenile and adult shoots from the progeny of a genetic cross between the olive varieties ‘Picual’ and ‘Arbequina’. Libraries were designed to contain RNAs with the size and the biochemical signatures (5′ phosphate and 3′ hydroxyl groups) of DCL cleavage products. A total of 485,108 unfiltered reads were obtained from both libraries, of which 195,149 and 289,959 sequence reads corresponded to juvenile and adult shoots, respectively (Table 1). After removing possible artifacts including products of multiple adapter ligations or empty constructs without a sRNA, 191,257 and 280,959 sequences with recognizable flanking adapter sequences remained for further analysis. These reads represented 89,945 and 66,978 unique sequences from juvenile and adult shoots, respectively. The length distribution of the sRNA sequences ranged from <20 nts to >25 nts (Figure 1). Surprisingly, sequences larger than 25 nts were the most abundant species in adult shoots when the total number of unfiltered reads was considered (Figure 1A). Blast analysis against publicly available plant repeat databases revealed that they were mostly contaminants of non-DCL-dependent RNAs such as degradation products of non-coding RNAs (rRNA, tRNA, snRNA and snoRNA) and several classes of transposons, Indeed, there was a near-equal size distribution in all size classes longer than 25 nts suggesting that these were not siRNAs (Figure 1B). We were aware though that some of these RNAs were, or could be, authentic siRNAs such as those derived from rDNA [6]. For instance, 21–22 nt sRNAs that hit rRNA-coding genes could be found in our Blast analysis. Interestingly, some of these sequenced rRNA-derived sRNAs matched regions with the potential of forming stem-loop-like structures, suggesting the possibility that such loci might generate miRNA-like sRNAs (Figure S1A). However, using a PCR approach that used the RT-PCR-amplified sRNA libraries as a template [42] we detected the expression of additional sRNAs throughout the rRNA precursor (Figure S1B). Primers were designed to detect sRNAs from the stem region as well as from outside of the predicted foldback within the rRNA transcript. To assess the possibility that the generated PCR products resulted from adventitious primer amplification, the PCR products were cloned and sequenced. Sequencing analysis corroborated the identities of the amplified products and revealed length variants with 5′ polymorphisms (data not shown) that were fully consistent with flexible DCL cleavage events of the rRNA precursor. We concluded that these rRNA-derived sRNAs, which were missed in the sequenced set, were due simply to RNA silencing rather to specific processing of the structured precursor or RNA fragmentation.

**Table 1:** Summary of sequencing results from juvenile and adult shoots of *O. europaea*.

	Total		Unique	
	Reads	Matching olive cDNA*	Reads	Matching olive cDNA*
Juvenile shoots				
Raw data	195,149			
Adapter removed	191,257		89,945	
Filtered data	105,794	11,730	71,824	4,840
Match known miRNAs**	204		44	
Adult shoots				
Raw data	289,959			
Adapter removed	280,927		66,978	
Filtered data	63,905	6,109	47,408	3,309
Match known miRNAs**	22		15	

*Olive database contains 288.595 cDNA contigs and singletons.

**all possible miRNA sequence polymorphisms are counted.

Next, a set of three computational filters was applied to remove species outside the 18–25 nt size range (typical size range for DCL-derived products), species with low sequence complexity (less than 3 different bases), and species with non-coding RNA matches. A final subset of 105,794 (71,824 unique sequences) and 63,905 (47,408 unique) silencing sRNA reads from juvenile and adult shoots, respectively, was obtained and used in the analysis presented here (Table 1). Although some sRNAs were highly abundant in the data set, the vast majority of sequences (80%) were singletons suggesting that our sRNA libraries were far from saturated and that, consequently, olive contained a large and diverse sRNA population.

The overall size distribution patterns of total and unique sRNAs from both libraries showed striking similarities (Figure 1C). sRNAs in the range of 23 to 25 nts accounted for 96% of the total sequences, and reads of 24 nts represented the most prominent size class (80% of total) in both tissues analyzed. The 24-nt unique sequences also accounted to approximately 80% of the olive sRNA transcriptome compared, for instance, to the 60% observed for *Arabidopsis* [43]. The 24-nt class exhibited high sequence diversity consistent with the widespread origins of sRNAs of this size along plant genomes- out of 85,428 sequences that were 24 nts long in juvenile shoots, 57,644 unique sequences were found (67% of the 24-nt reads) while 38,352 sequences were unique among the 52,008 reads of 24 nts from adult shoots (73% of the 24-nt reads) (Figure 1C). Unprecedentedly, we barely counted 913 (526 unique) and 494 (361 unique) 21-nt sRNA reads in juvenile and adult shoots, respectively, which on average represented an unusual ∼0.8% of the sRNAs in the sequenced set (Figure 1C). Comparatively, the number of total and unique 24-nt species relative to the 21-nt class was about two orders of magnitude higher in both libraries. The representation of 22-nt RNA was also slightly higher than the 21-nt class: 1,398 (1,041 unique) and 784 (621 unique) 22-nt counts were found in juvenile and adult shoots, respectively (∼1.3% of the reads). The distribution of lengths for the set of redundant and non-redundant sequences was virtually identical in these two independent libraries precluding technical artifacts during amplicon preparation (Figure 1C). Furthermore, we do not think that the sequencing procedure biased in detriment of the 21-nt class as other 454-based sequencing initiatives for sRNA profiling in other flowering plants revealed a much larger representation of the 21–22 nt component relative to the 24-nt class [44]–[46]. To identify the potential genomic sources of olive sRNAs, all sequences were aligned using BlastN to the olive cDNA database (https://chirimoyo.ac.uma.es/oleagen/). However, 93.7% of 20 to 24-nt sequences passing the above filters could not be assigned to specific genomic loci. Presumably this is because the dominance of the 24-nt class, which was predicted to derive from genomic loci that are not represented in cDNA collections.

### Identification of conserved miRNAs in olive

Currently, miRNAs from about 24 broadly conserved families have been identified from eudicots to basal plants and deposited in the public available miRNA database miRBase [47]. Many other known non-conserved miRNA families in miRBase are species-specific or restricted to certain plant families. We followed a homology-based approach to search for already known miRNAs in our two olive libraries using miRBase (Release 17.0) as a reference set. Direct BlastN comparison identified 226 sRNAs of 18–22 nts in our sequenced collections that exhibited perfect or near perfect matches with at least 18 families of known miRNAs in the Viridiplantae, of which 17 and 11 families were identified in juvenile and adult shoots, respectively (Table 2). Candidate miRNA reads were usually 21-nt long, although variants with 5′ or 3′ heterogeneity such as shorter/longer RNA species or species that contained mismatches at the 5′ or 3′ terminus (allowing up to two mismatches) were also detected. For instance, the family of oeu-miR159 (136 reads) had eleven variants in the dataset of which five contained internal mismatches with respect to the most frequently observed sequence (113 reads) and six were length variants that deviated by one to three bases at their ends. sRNAs with perfect matches with the complementary miRNA* strands of miR166, miR168 and miR396 were detected in our dataset. cDNA mapping was, in most cases, impractical likely due to the incompleteness of the olive genome, and pre-miRNAs holding their characteristic secondary structures could only be predicted for oeu-miR159, oeu-miR166, oeu-miR167, oeu-miR169, oeu-miR172 and oeu-miR390 (Figure S2). As a result, the polymorphic sequences identified here as miRNAs could not be assigned to a particular *MIRNA* gene because it was not possible to indubitably assure whether they were different members of a family or variants of the miRNA produced from the same precursor (Table 2).

**Table 2:** Sequence and length polymorphisms for known miRNAs in *O. europaea*.

miRNA family	Sequence (5′-3′)	Size	Juvenile	Adult
miR156/157	UUGACAGAAGAUAGAGAGCGC	21	N	1
miR159	UUUGGAUUGAAGGGAGCUCUA	21	109	4
	UUCGGAUUGAAGGGAGCUCUA	21	1	0
	UUUGGAUUGAAGGUAGCUCUA	21	1	0
	UUGGUAUUGAAGGGAGCUCUA	21	1	0
	UUUGGAUUGAAGGGAGCUUUA	21	2	0
	UUUUGGAUUGAAGGGAGCUCUA	22	1	0
	CUUUGGAUUGAAGGGAGCUCUA	22	1	0
	UUUGGAUUGAAGGGAGCUCUAA	22	1	0
	UUUGGAUUGAAGGGAGCUCU	20	4	0
	UUGGAUUGAAGGGAGCUCUA	20	3	0
	UUUGGAUUGAAGGGAGCU	18	8	0
miR160	UGCCUGGCUCCCUGUAUGCCA	21	3	2
miR164	UGGAGAAGCAGGGCACGUGCA	21	8	1
	UGGAGAAGCAGGGUACGUGGA	21	2	0
	UGGAGAAGCAUGGCACGUGCA	21	1	0
miR166	UCGGACCAGGCUUCAUUCCCC	21	1	0
	UCGGACCAGGCUUCAUUCCUC	21	2	0
	UCUCGGACCAGGCUUCAUUCC	21	1	0
	UCGGACCAGGCUUCAUUCC	19	0	1
	UCGGACCAGGCUUCGUUC	18	0	2
miR166*	GGAAUGUUGGCUGGCUCGAGG	21	1	0
	GGAAUGUUGUCUGGCUCGAGG	21	0	1
	GGAAUGUUGGCUGGCUCGAGGC	22	1	2
miR167	UGAAGCUGCCAGCAUGAUCUA	21	2	1
	UGAAGCUGCCAGCGUGAUCUA	21	1	0
	UGAAGCUGCCAGCAUGAUCUGG	22	2	0
	UGAAGCUGCCAGCAUGAUC	19	13	1
	UAAAGCUGCCAGCAUGAU	18	1	0
miR168	UCGCUUGGUGCAGGUCGGGAA	21	7	1
	CGCUUGGUGCAGGUCGGGAAC	21	1	0
miR168*	CCCGCCUUGCAUCAACUGAAU	21	1	0
miR171	UGAUUGAGCCGUGCCAAUAUC	21	1	0
miR172	AGAAUCUUGAUGAUGCUGCAU	21	1	0
	AGAAUCCUGAUGAUGCUGCAU	21	1	0
miR390	AAGCUCAGGAGGGAUAGCGCC	21	0	1
	AGCUCAGGAGGGAUAGCGCC	20	2	0
miR396	UUCCACAGCUUUCUUGAA	18	4	0
miR396*	GUUCAAGAAAGCUGUGGGACA	21	1	0
miR482	UCUUACCAAUGCCUCCCAUCCC	22	1	0
	UUUCCUAUUCCUCCCAUACCGA	22	0	2
miR845	AGGCUUUGAUACCACUUG	19	2	0
miR858	UUCGUUGUCUGUUCGACCUUA	21	3	1
miR894	UGUUUCACGUCGGGUUCACCA	21	1	0
miR1310	GAGGCAUCGGGGGCGCAA	18	1	0
miR2911	GCGGCCGGGGGACGGACUGGG	21	1	0
	CUGGCCGGGGGACGGACUGGGA	22	1	0
	GUGGCCGGGGGACGGACUGGGA	22	1	0
	UCGGCCGGGGGACGGACUGGGA	22	0	1
	GCCGGGGGACGGACUUGGA	19	2	0
miR4342	CUAAGGAUGUAGGGUGGU	18	1	0

The number of times a sequence was sampled in juvenile and adult shoots is indicated. (N) denotes miRNAs detected by Northern blot hybridization using sequence-specific probes. Up to three mismatches were allowed with respect to the canonical miRBase sequence.

### Differential expression of conserved miRNAs during olive development

We investigated the expression profile of some olive miRNAs in juvenile and adult shoots, in growing and dormant lateral buds and in different organs from different genetic backgrounds under the assumption that differential expression patterns could be diagnostic of a developmental- and/or tissue-specific biological function [48], [49]. First, we exploited high-throughput sequencing data from our ‘Picual’×‘Arbequina’ libraries to infer miRNA relative abundance in juvenile and adult developmental stages. In our study, the majority of known miRNAs were sequenced less than 5 times in each sample and some of them were retrieved only once in the dataset (Table 2). As a result, differences in the expression profiles of these miRNAs between juvenile and adult shoots were unreliable. In contrast, a few other miRNA families had at least 10 read counts in one of libraries, and were used for quantitative comparison of abundance. For instance, after count normalization (reads per thousand; rpt), oeu-miR159, the most frequent miRNA in our two sequencing datasets, was clearly overrepresented in juvenile tissue (1.15 rpt) compared to adult shoots (0.06 rpt) (20∶1 ratio). Also, oeu-miR167, oeu-miR168 and oeu-miR164 exhibited a moderate increased in their sequencing frequencies in juvenile *versus* adult shoots (about 10∶1 ratio).

**Figure 2:**
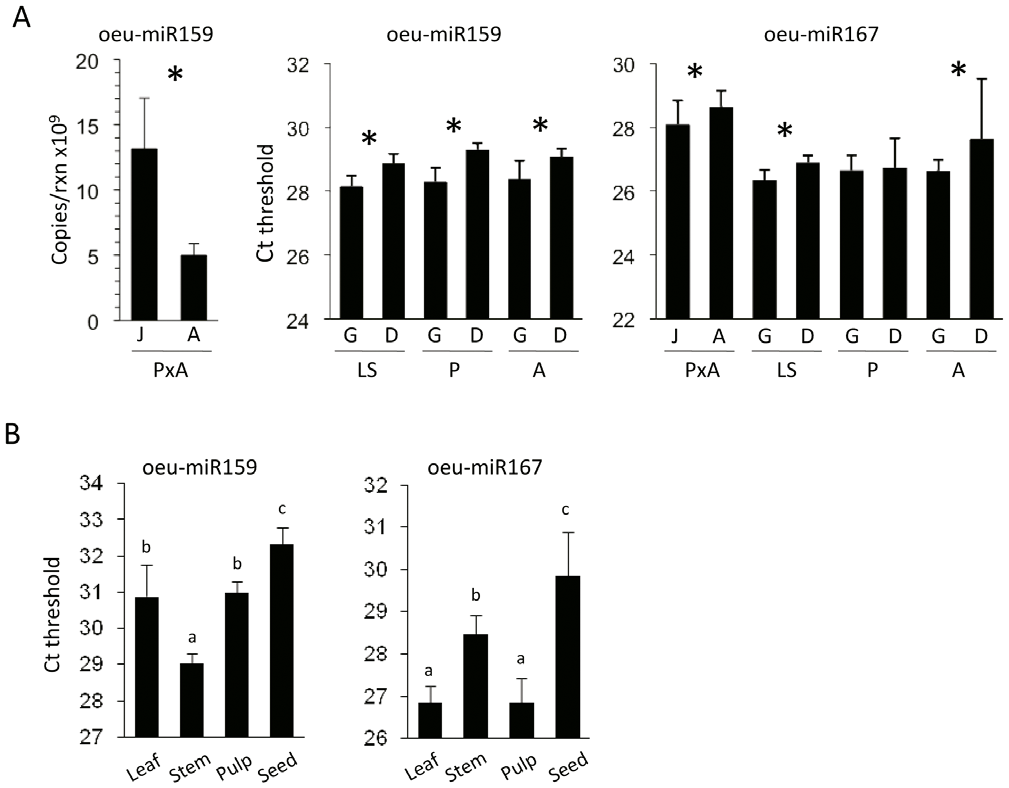
Expression analysis of representative olive miRNAs using quantitative stem-loop RT-PCR. Expression of miR159 and miR167 was quantified in juvenile (J) and adults (A) shoots as well as growing (G) and dormant (D) buds from different olive genotypes (*A*) and in different olive tissues (*B*). miRNA abundance is expressed as the number of copies per reaction (absolute quantification method) or as function of the threshold cycle (Ct) as indicated. Note that the Ct is inversely proportional to the relative abundance of each miRNA. Error bars indicate standard deviation of three different technical repeats of each of two biological replicates. Significant differences at P<0.01 (One Way ANOVA and Duncan test) between samples are indicated with asterisks or with different letters. Olive varieties as follow: (P) ‘Picual’, (A) ‘Arbequina’, (LS) ‘Lechin de Sevilla’, (PxA) ‘Picual’×‘Arbequina’ cross.

Because sequencing abundance does not necessarily correlate with *in vivo* abundance [13], we chose oeu-miR159 and oeu-miR167 as representatives to investigate their expression using stem-loop quantitative RT-PCR. To strengthen the robustness of our experiment and minimize the disturbing effect of sample variability, RNA samples from juvenile and adult shoots of the ‘Picual’×‘Arberquina’ cross, previously used for library construction, as well as total RNA from growing (active) and dormant lateral buds from other olive varieties (‘Picual’, ‘Arbequina’, ‘Lechín de Sevilla’) were tested. The results of the qRT-PCR reactions showed that oeu-miR159 and oeu-miR167 were expressed in all developmental stages and that each reproducibly accumulated to higher levels in juvenile and growing tissues relative to adult and dormant tissues in their respective genotypes (Figure 2A). These results experimentally corroborated that the sequencing frequencies, at least for the most predominant miRNA species, were good indicators of miRNA abundance in olive tissues.

**Figure 3:**
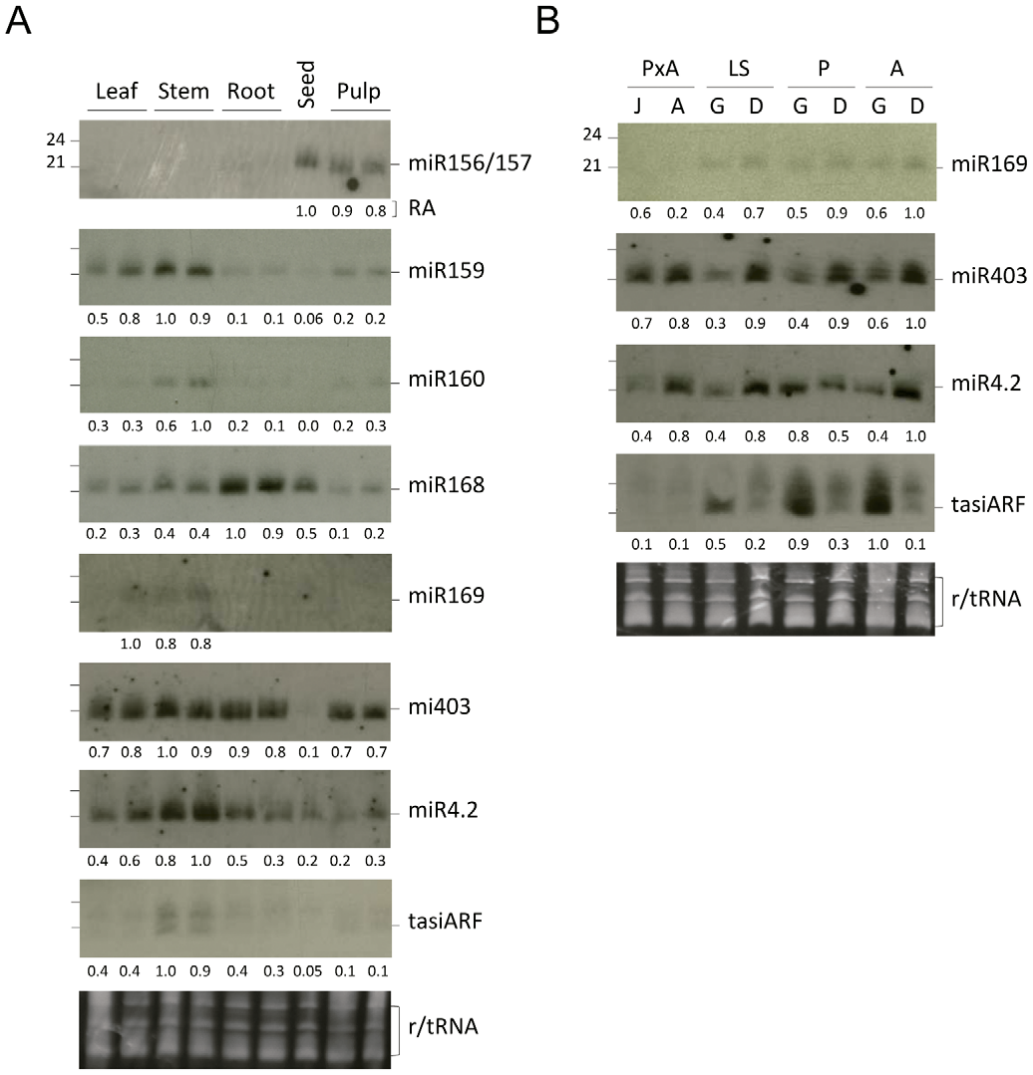
Expression patterns of selected known miRNAs in *O. europaea*. RNA preparations from single or duplicated samples obtained from different tissues (*A*) and developmental stages (*B*) were analyzed by Northern blots using radiolabeled-oligonucleotide probes complementary to each miRNA sequence. Numbers below each panel refer to accumulation levels (RA) relative to the sample with the highest hybridization signal (arbitrarily designated as 1.0). Ethidium bromide-stained RNA (prior to transfer) is shown as loading control. Membranes were stripped and reprobed. (J) juvenile shoots, (A) adult shoots, (G) growing buds, (D) dormant buds. The following olive varieties were used: (P) ‘Picual’, (A) ‘Arbequina’, (LS) ‘Lechin de Sevilla’, (PxA) ‘Picual’×‘Arbequina’ cross.

To examine tissue-specific expression of olive miRNAs from our sequence set, we performed stem-loop RT-PCR and northern blot assays of RNA samples from leaves, roots, stems, pulps and seeds. Based on the threshold cycle (Ct), oeu-miR159 was highly expressed in stems compared to leaves and pulps (Figure 2B). Contrarily, oeu-miR167 was expressed at a higher level in leaves and pulps than in stems (Figure 2B). Both miRNAs had much lower expression levels in olive seeds. Northern analysis of duplicated samples confirmed the expression pattern of oeu-miR159 and revealed that oeu-miR160 was also preferentially expressed in stems relative to leaves, roots, pulps and seeds, whereas oeu-miR168 was particularly abundant in root tissue (Figure 3A). sRNA blot assay showed that oeu-miR156/157 in the form of 21 nts was expressed at higher levels in pulp, whereas it accumulated as a unique band of 22 nts in seeds, suggesting different DCL targeting specificities for the miR156/157 precursor during olive development (Figure 3A).

Several additional miRNAs that were absence from our libraries were also selected for developmental stage- and tissue-specific analysis by sRNA hybridization assay. We found that oeu-miR169, that was below detection limits in samples from juvenile and adult shoots, accumulated as a discrete size band of ∼21 nts at comparable weak levels in growing and dormant lateral buds, and was slightly more profuse in leaves and stems than in roots, pulps and seeds (Figure 3A,B). Oeu-miR403 was similarly rich in all tissues interrogated (but seeds), it exhibited a nearly equal abundance in juvenile and adult shoots, and was particularly abundant in dormant buds compared to growing buds in all olive varieties tested (Figure 3A,B). We failed to detect miR158, miR395 or miR398 in olive tissues and developmental stages using sequence-specific radiolabeled probes complementary to their counterparts in *Arabidopsis* (data not shown). In summary, the data confirmed that the expression and accumulation of certain miRNAs is tissue-specific and it is subjected to developmental control.

### Prediction of novel olive miRNAs

To uncover additional olive-specific miRNA candidates within our sequenced set, a total of 104,501 unique sRNA species of 20 to 24 nts was selected from our combined libraries for further analysis. Sequences were aligned against the *O. europaea* cDNA collection in order to identify sRNA-containing cDNA contigs that may serve as putative precursors for miRNA biogenesis. Comparison analysis identified 6,598 unique olive sRNAs with perfect alignments to 3,403 olive cDNAs (1,453 contigs and 1,950 singletons). Secondary structure analysis predicted 5 possible miRNAs deriving from cDNA sequences with intramolecular folding capacity that satisfied established secondary structure criteria (see material and methods section) (Table S1, Figure S3). Each of these potential miRNAs represented a distinct family, and one of the hairpin-like sequences was predicted to produce two different miRNAs (oeu-miR4.1 and oeu-miR4.2), each one arising from different arms of the stem-loop (Figure S3). None of these 5 putative miRNAs was supported by a miRNA* in the sequenced list, making their classification as miRNAs questionable. This observation was not surprising due to the non-saturating coverage of our sRNA libraries and the fact that non-conserved miRNAs and their miRNA*s are generally expressed at low levels or in specific cell-types or growth conditions [13]. RNA blots showed that miR4.2 tended to accumulate in dormant relative to growing buds and exhibited ubiquitous expression in all tissues tested (seed, pulp, root, leaf and stem) although it was more abundant in stems (Figure 3). Hybridization signals for the rest of candidate miRNAs were not detected (data not shown). Finally, BlastN analysis against all nucleotide sequences in the NCBI databases revealed that no homologues for these 5 sRNAs were found in other plant species, suggesting that these newly identified putative miRNAs were olive-specific.

### Target prediction and miRNA-guided cleavage analysis

To gain insights into the functions of known and novel miRNAs in olive, putative target genes of olive miRNAs were computationally predicted among the bulk of olive cDNA sequences using a penalty/scoring-based method (see material and methods section) [50]. As a result, all olive miRNAs interrogated had at least one predicted target gene (some miRNAs had multiple targets) with a score of up to 3.5 among the cDNA collection, although many others may be not yet represented in the olive dataset (Table 3). All olive cDNAs predicted to be targets of known miRNAs were orthologues of miRNA target genes in *Arabidopsis* and other plant species. Interestingly, Apetala 2 (AP2) and Squamosa Promoter Binding Protein-Like (SPL) coding genes were identified as targets of miR172 and miR156, respectively, suggesting that these two miRNA nodes are likely operative during developmental transitions in olive [51], [52].

**Table 3:** Predicted olive cDNA targets for known olive miRNAs.

miRNA	Predicted olive cDNAs	Score	Blast TAIR (Gene products)
oeu-miR157	contig #38893.6	1	SQUAMOSA PROMOTER BINDING PROTEIN-LIKE 10 (SPL 10) (At1g27370.4)
	contig #84769.6	1	SPL 10 (At1g27370.4)
	contig #37712.6	2	SPL 13 (At5g50570.2)
oeu-miR159	contig #34844.6	1.5	MYB DOMAIN PROTEIN 104 (MYB 104) (At2g26950.1)
	contig #44056.6	1.5	MYB 104 (At2g26950.1)
	contig #110183.6	2	MYB 33 ( At5g06100.3)
oeu-miR160	contig #48039.6	2	AUXIN RESPONSE FACTOR 10 (ARF 10) (At2g28350.1 )
oeu-miR164	contig #21928.6	1	NAC DOMAIN PROTEIN 22 (At1g56010.2)
	contig #79896.6	1	NAC DOMAIN PROTEIN 80 (At5g07680.2)
	contig #44877.6	2	NAC DOMAIN PROTEIN 80 (At5g07680.2)
	contig #27768.6	3	NAC DOMAIN PROTEIN 2 (At5g39610.1)
oeu-miR166	contig #35489.6	2	ATHB 15 (At1g52150.3)
oeu-miR167	contig #28070.6	1	ARF 8 (At5g37020.2)
	contig #45795.6	1	ARF 8 (At5g37020.2)
oeu-miR168	contig #26385.6	1.5	ARGONAUTE 1 (At1g48410)
oeu-miR169	contig #57460.6	1.5	NUCLEAR FACTOR Y, SUBUNIT A9 (At3g20910.1)
oeu-miR171	contig #59526.6	0.5	GRAS DOMAIN PROTEIN (SCARECROW-like) (SCL) (At2g45160.1)
	contig #66427.6	0.5	SCL (At3g60630.1)
oeu-miR172	contig #33506.6	1.5	APETALA 2 (At4g36920.1)
	contig #61131.6	1.5	APETALA 2 (At4g36920.1)
	contig #15488.6	2.5	APETALA 2 (At4g36920.1)
oeu-miR390	contig #45914.7	3.5,4	TAS3 (At3g17185)
	contig #48241.7	3,4	TAS3 (At3g17185)
oeu-miR482	contig #81599.7	3	DISEASE RESISTANT PROTEIN (At5g45240.1)
oeu-miR858	contig #97262.7	2.5	MYB 83 (At3g08500.1)
oeu-miR894	contig #65792.7	3	YPT/RAP GAP DOMAIN SUPERFAMILY PROTEIN (At3g59570.1)
tasiARF	contig #18473.7	1,1.5	ARF 4 (At5g60450.1)
	contig #49012.7	1,1.5	ARF 4 (At5g60450.1)
	contig #83145.7	1,1.5	ARF 3 (At2g33860.1)

Versions 6 and 7 of the olive cDNA database were used for Blast search. The origin of each contig is indicated accordingly. Scores calculated using psRNATarget (http://bioinfo3.noble.org/psRNATarget/index.php) as suggested by [50].

**Figure 4:**
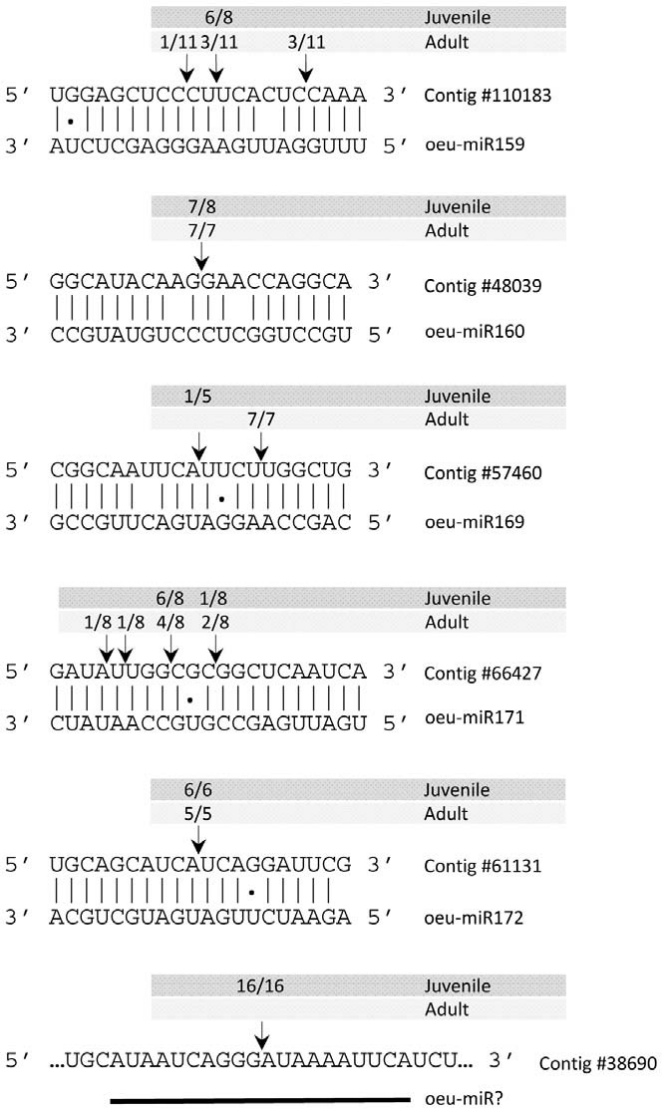
Experimental validation of the predicted mRNA targets of oeu-miRNAs. miRNA-guided cleavage sites were identified by RLM-5′ RACE using total RNA preparations from juvenile and adult shoots. Arrows indicate mapped cleavage positions with the frequency amongst clones sequenced. Target cDNA sequences are shown on top of the miRNA sequences. Olive contig #38690 was identified as a putative sRNA target (see text).

In plants, miRNAs interact with target transcripts to promote AGO1-mediated slicing near the middle of the base pair interaction region [12], [53]. miRNA-guided, sequence-specific endonucleolytic cleavage events can be identified *in vivo* using RNA ligase-mediated 5′ rapid amplification of cDNA ends (RLM-RACE) [54]. In this study, 5′ RACE assays were done using RNA preparations from juvenile and adult shoots and gene-specific primer sets. Cleavage sites in five of the predicted target genes of conserved miRNAs (oeu-miR159, oeu-miR160, oeu-miR167, oeu-miR171, and oeu-miR172) could be identified (Figure 4). For the rest of the conserved miRNA/cDNA pairs tested, we failed to amplify a major PCR product as diagnostic of miRNA-directed cleavage (data not shown). Sequencing of 5′ ends revealed cleavage events directed by oeu-miR160 and oeu-miR172 at a predominant position at the centre of the miRNA/mRNA interaction (position 10 to 11) in both tissues analyzed (Figure 4). However, oeu-miR159 and oeu-miR171 yielded a predominant cleavage site within the complementary region in juvenile tissues, whereas there were several and minority cleavage sites distributed along the miRNA/mRNA duplex in adult shoots. In the case of oeu-miR169, a predominant cleavage site was only detected in adult tissues (Figure 4).

cDNA contigs containing imperfect but extensive complementarity with potentially novel miRNAs were predicted as targets (Table S2). We were, however, not successful in identifying miRNA-guided cleavage activity on the predicted targets and, therefore, these miRNAs could not be confirmed as functional. Surprisingly, 5′ RACE experiments for validation of contig #38690 as a target of oeu-miR4.1 revealed a unique amplification product from which 5′ end sequencing identified a dominant and reproducible cleavage site (16/16 clones sequenced) located 11 nts upstream from the predicted oeu-miR4.1 binding site (Figure 4). The fact that a single PCR product was obtained along with the precise mapping reproducibility suggested that this 5′ end represented an authentic sRNA-cleavage site and not a random cleaved end from fragmented RNA. We failed to identify a sRNA in our sequenced set with near-perfect complementarity to the corresponding binding site, likely due to the low sequencing coverage of our libraries.

### Silencing networks for developmental timing in olive

**Figure 5:**
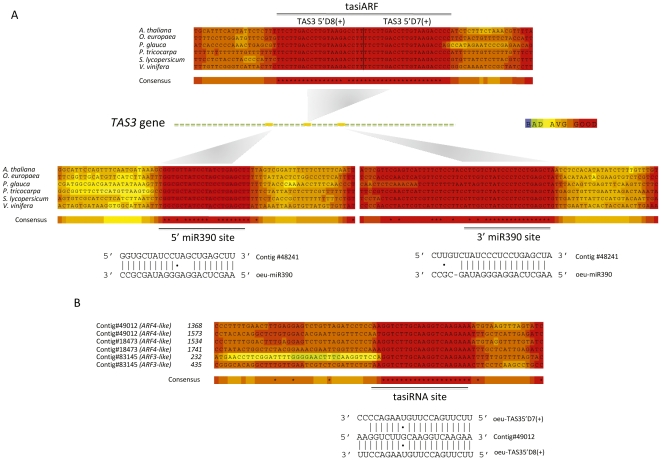
Identification of tasiARF-containing *TAS3* olive genes and putative ARF target sequences. (*A*) Schematic alignment of predicted transcripts and ESTs for *TAS3* genes (tasiARF precursors) from olive and other seed plants. At least two olive cDNA clones (contig #48241 was shown as a representative) contain dual miR390 complementary sites that flank the area of predicted tasiRNA production. The regions corresponding to the 5′ miR390 complementary site, tasiARFs, and the 3′ miR390 complementary site are expanded. tasiARF refers to the regions homologous to Arabidopsis TAS3 5′D7(+) and TAS3 5′D8(+). miR390 binding sites are indicated. (*B*) Alignment of *ARF-like* target genes from olive in the regions of sequence complementarity to tasiARF. Three putative ARF contigs in the olive database are shown to contain each two separated tasiARF complementary sites. Alignments and color-coded based on the confidence of the local alignment were generated using T-Coffee and its CORE function.

ARF-interacting tasiRNAs derived from the *TAS3* locus, namely tasiARFs, control proper timing of vegetative phase transitions through negative regulation of mRNAs encoding ARF2, ARF3 and ARF4 [55]–[59]. Since an important goal of our study was to unmask sRNAs controlling developmental transitions, we conducted an exhaustive Blast search using Arabidopsis *TAS3* genes as queries to find putative *TAS3* genes in the olive database [50], [60]. Three olive cDNA contigs were found to contain discrete sequences similar to *TAS3* genes and complementary to *ARF* genes. Contigs #48241 and #45914 had both two near-identical 21-nt sequences adjacent to one another of which one was identical to Arabidopsis tasiARF at phase 5′D7(+) and the other contained a single mismatch with respect to the Arabidopsis tasiARF at position 5′D8(+) (Figure 5A). Contig #63271 also contained two tasiARF sequences with 3 and 1 mismatches relative to the Arabidopsis 5′D7(+) and 5′D8(+) counterparts, respectively (Figure S4). As observed previously in other plant species [24], [50], [60]–[62], we did not detect any sequence similarity between all putative *TAS3* genes from olive and other plant genomes interrogated in this study outside of the region containing the candidate tasiARFs (Figure 5A). The *TAS3* family is distinguished from other *TAS* loci by the dual miR390 binding sites, which are functionally required by *TAS3* mRNA to define the phasing register for tasiRNA production [50], [62], [63]. Two miR390 recognition sequences flanking the tasiARF region were observed in the putative olive *TAS3* transcripts represented by contigs #48241 and #45914 (Figure 5A). The cDNA contig #63271 was shorter than the full-length *TAS3* mRNA precursor and miR390 sites could not be mapped. Therefore, the presence of miR390 complementary sites in these olive *TAS3* loci supported the idea that they were indeed tasiRNA genes, and suggested that miR390 set the phasing register of olive tasiARFs [50], [62]. Indeed, tasiARF sequences in contigs #48241 and #45914 also coaligned with the phases D7(+) and D8(+) defined by the 3′ miR390 processing site in their putative precursors as observed in moss and angiosperms [50], [62].

Whereas miR390 expression in olive was confirmed through deep-sequencing, none of the two predicted olive tasiARFs was detected in our sequenced libraries and therefore we studied tasiARF accumulation in olive tissues by Northern blot. A LNA (locked nucleic acid) probe was necessary to reveal hybridization signals in the form of two discrete bands of ∼21 and 24 nts that were weak and equally abundant in juvenile and adult shoots (Figure 3B). In contrast, tasiARFs accumulated abundantly in growing buds to levels higher than in dormant buds in the three genotypes tested, which agrees with their role as modulators of developmental timing [52]. tasiARF were more prominent in stems than in other tissues tested (Figure 3A,B). In conclusion, our data revealed three candidate genomic loci for the tasiARF-containing TAS3 precursors supported by their corresponding ESTs in the olive database, of which at least two contained dual miR390 binding sites and can be regarded as tasiARF-generating TAS3 precursors [62].

Furthermore, we also identified in the olive cDNA database homologues of the Arabidopsis *ARF3* and *ARF4* genes as potential tasiARF targets as they shared a 21-nt region of sequence complementarity to the olive tasiARFs (Figure 5B). *ARF4-like* mRNAs, represented by contigs #18473 and #49012, and *ARF3-like* mRNA, represented by contig #83145, possessed each two TAS3-pared recognition sites (Figure 5B) as observed in other land plants [50], [60], [62]. Although 5′ RACE could not confirm tasiARF-mediated cleavage of the *ARF3* and *ARF4* target transcripts, our data collectively confirmed that the tasiARF regulatory node is broadly conserved in the plant kingdom and suggested that it is fully operational during developmental timing in the olive vegetative growth.

## Discussion

In this study we used deep pyrosequencing supported by conventional RNA methods to identify and characterize miRNAs from different developmental stages and tissues in several commercial olive genotypes. This work further provides the first draft of the olive sRNA transcriptome and reinforces the notion that the sRNA component is extraordinarily complex and diverse within the plant kingdom. sRNA profiling using high-throughput sequencing is, in most cases, straightforward for model plants for which genomic and biocomputing tools are widely implemented [6], [43], [64]. sRNA analysis is however challenging for plant species such as olive for which the genome is largely unknown. This situation is aggravated by the size of the olive genome, four times the size of the poplar genome (a model plant for trees) and eight times the size of the *Arabidopsis* genome [39], [65]. As a result, limited genome information imposed a major constraint for sRNA annotation and identification of the many sources of olive sRNA production, including *in silico* predictions, that restricts our knowledge on its origin and biosynthetic pathways.

Excluding low quality reads and abundant RNA products derived from degradation of non-coding RNA, which accounted for about 40% of the total unfiltered reads, the size distribution pattern of olive silencing sRNAs was consistent with DCL processing events but deviated significantly from that expected. Indeed, a hallmark signature of the olive sRNA population is the overwhelming presence of 24-nt species at a level higher than that observed in many other plant species [13], [18], [38], [66]. This was not due to possible redundancies in the 24-nt population since the ratio of redundant and non-redundant sequences was comparable to other size classes. Only 5.2% of the 24-nt sRNAs perfectly mapped to the olive cDNA collection as opposed to the 13% of the 21-nt sRNA class (hits normalized with respect to the size class with the lowest number of unique counts), supporting the prediction that 24-nt species should originate preferentially from heterochromatin and intergenic regions in the olive genome. Furthermore, the olive 24-nt pool was enriched for a 5′ terminal adenosine (A) (nearly 60% of the unique counts of this size), just as the 24-nt class of heterochromatin-associated siRNAs [6], [13], [67]. Preference for a 5′ terminal A predicts a potential association to AGO4, the AGO operating during transcriptional gene silencing, as observed for DCL3-dependent, 24-nt siRNAs in *Arabidopsis* [68], [69]. Although this size class is consistent with DCL3 processing of dsRNA precursors [6], genetic studies to correlate the generation of endogenous sRNAs with specific DCL or RDR activities are not possible in olive yet. Because siRNAs of 24 nts normally guide sequence-specific methylation events at the DNA and/or chromatin level [1], [27], our data suggests that epigenetic control and re-programming of heterochromatic elements by siRNAs may be critical for genome stability in olive. This reasoning is logical since the high genome size of *O. europaea* is likely accompanied by increasing amounts of noncoding repetitive sequences. Also, the overrepresentation of the 24-nt size class in olive likely reflects specific necessities to maintain and organize its sized genome during extended juvenile phase, seasonal senescence and dormancy/growth arrest.

Perhaps, the most distinguishing property of the sequenced set is the comparatively low abundance of the 21-nt class with respect to other plant species. Furthermore, the miRNA class accounted for only a residual fraction within the pool of sRNAs of 19 to 22 nt (5.7% and 1.1% in juvenile and adult shoots, respectively). This finding sharply differed from that seen in sRNA libraries obtained through deep-sequencing from other plant species where reads of 21 nts (mostly miRNAs) were among the most abundant sRNA species [6], [13], [45], [49], [66], [67], [70]–[72]. For instance, sRNAs of 21 nts (followed by 22 nts) dominate the sRNA scene in other wooden plants such as conifers, which lack DCL3 activity, grapevine, or poplar [38], [71], [73]–[75]. The high level of miRNAs in cultivated woody plant has been hypothesized to be a consequence of their highly heterozygous genomes, a prediction that does not fit with our observation in cultivated olives [18]. In general, the unusual underrepresentation of 20 to 22-nts in both olive sRNA libraries added more difficulties to profiling the miRNA component of the olive transcriptome. Under this evident constraint, it is also due to depth of sequencing that our sequenced set contained relatively few miRNAs compared to other recent sequencing-based initiatives and that most of the identified miRNAs did not have either a substantial number of reads in the sRNA libraries [18], [66]. Deeper sequencing, or even alternative sequencing platforms, could give better resolution in the olive sRNA population, therefore unraveling more microRNAs. In addition, several other factors that include the genome size of the plant, or the developmental stages at which samples were collected for deep sequencing may explain this situation.

*MIR156/157, MIR160, MIR164, MIR168, MIR171, MIR396, MIR403, MIR482, MIR845, MIR858, MIR894, MIR1310, MIR2111* and *MIR4342* family members were annotated based on their phylogenetic distribution, as due to the lack of genomic resources their loci could not be identified in our study. In addition, other sRNAs, not supported by their star strands, were considered as novel miRNA candidates as they presumably derived from regions with the potential to fold into stem-loop structures. Further work is needed to determine whether these loci produce a functional miRNA/miRNA* duplex or they represent false positives. We presumed that more olive-specific miRNAs still await discovery provided that deeper sequencing efforts are carried out and that a comprehensive olive EST repository is available.

Our repertoire of known miRNAs revealed notorious sequence heterogeneity at the ends of the miRNAs. Although the most frequent sequence for each known miRNA was often identical in composition and length to the mature miRNA sequence in the miRBase, variants that possessed altered internal nucleotides or shortened or lengthened 5′ or 3′ ends were found. It is very probable that positional variants reflect different members within a family while sequences shorter than the reference miRNA could account for degradation products of the canonical miRNA. However, miRNA variants with lengths longer than the miRBase sequences (unlikely to be degradation products) were also retrieved. These length variants might result from i) suboptimal cleavage within a given pre-miRNA owing to occasional DCL slippage or flexible DCL1 targeting, or ii) independent processing of the same miRNA precursor by DCL2, DCL3 or DCL4. Although genetic tools to test this latest hypothesis are not available in olive, this observation is well documented in other plant species [18], [76]. Nevertheless, other scenarios for length heterogeneity should be considered such as sequencing errors and/or downstream processing that remove terminal nucleotides of the mature miRNA,

Gene targets were computationally predicted for both conserved and candidate novel miRNAs, although only target genes for a few conserved miRNAs proved to be real targets. Validate targets had major cleavage sites that mapped between the 10^th^ and 11^th^ position from the 5′ end of the miRNA, although deviations from the canonical cleavage sites were also found likely due to the cleavage activity of polymorphic length variants. Other than technical failures, targets that gave negative results upon 5′ RACE cleavage analysis might be false positive predictions. Interestingly, a newly identified olive cDNA was experimentally validated through 5′ RACE analysis as a cleavable target, although the miRNA or tasiRNA responsible for cleavage could not be identified in our sequence set. As a result, we cannot fully discard that it could be a degradation product. Because most of the predicted targets of candidate miRNAs had no hits in other plant species, no definitive conclusions could be drawn about their biological functions, although functions might be olive-specific.

In this study, we identified three candidate *TAS3* genes through EST database mining in olive of which at least two contained two adjacent, nearly-identical tasiARFs and dual miR390 complementary sites. Expression of *ARF3* and *ARF4* mRNAs with two sites complementary to tasiARF 5′D7(+) and 5′D8(+) was also confirmed in olive tissues providing evidence that the miR390-TAS3 pathway is conserved in olive, likely to regulate phase transition [52], [77]. In addition, we show that genetic components of the miR156 and miR172 nodes are also conserved in olive [52], [77]. These two miRNA nodes promote progression through different developmental phases including transition from the juvenile to the adult stage of vegetative growth, and flowering [78], [79]. miR156 and miR172 target for negative regulation SPL and AP2-like transcription factors, respectively. SPL was computationally predicted as a target of oeu-miR156 while oeu-miR172-guided cleavage of olive *AP2* mRNA was experimentally validated in our study. In an antagonistic fashion, miR156 activity contributes to restrain development while miR172 functions to induce adult leaf features and flowering [52], [77]. In this interactive network, miR156 target SPL promotes the expression of miR172 by binding to the *MIR172* promoter, and consequently reduces the activity of AP2-like floral repressors targeted by miR172. Furthermore, miR172 target AP2-like proteins represses ARF3 expression by directly binding to the *ARF3* promoter, which in turn is controlled by tasiARFs from the *TAS3* locus. Besides ARF3 is presumably an activator of several SPL proteins in a miR156-independent fashion [52]. In conclusion, these findings provide new insights into the underlying mechanisms of juvenile-to-adult transition in olive trees.

## Materials and Methods

### Plant materials and RNA isolation

Olive samples were collected from the World Olive Bank of Germplasm (WOBG) at the IFAPA/UCO (“Alameda del Obispo”, Córdoba, Spain). Olive genotypes used in this study included the varieties “Lechin de Sevilla”, “Picual” and “Arbequina”, “Picual” and “Arbequina” exhibit different fruit and oil characteristics and tree architecture, and are the most broadly cultivated varieties in Spain, while “Lechín de Sevilla” shows intermediate phenotypes for all the above traits [80]. Growing (active) and dormant lateral bud samples were collected from the three above-mentioned cultivars. Several representative buds from each tree were sampled. Root, young leaves and stem samples were harvested from a single vegetative propagated adult tree of “Lechin de Sevilla”. Seeds and fruit mesocarp (pulp) were collected from olive fruits of “Picual” trees. Juvenile and adults shoots were collected from the segregating progeny (6 seedlings) of a breeding cross between “Picual” and “Arbequina”. After collection, all samples were immediately frozen in liquid nitrogen and stored at −80°C until used. Total RNA was extracted with the TRIZOL reagent (Invitrogen) following the manufacturer's instructions.

### sRNA library construction and sequencing

sRNAs libraries were prepared without cloning as described [46] using total RNA extracted from juvenile and adult shoots. RNA samples from 6 different olive trees were combined to form a single RNA pool from each developmental stage. Briefly, sRNA fractions of 15 to 40-nt long were purified by size fractionation with 15% polyacrylamide gels (PAGE) containing 8M urea followed by gel elution in 0.3 M NaCl and RNA precipitation. The isolated sRNAs were then sequentially ligated to adapters using T4 RNA ligase. A pre-activated 3′ adenylated oligo (5′ rAppCTGTAGGCACCATCAAT3ddC 3′) (Integrated DNA technologies) was used as a 3′ adapter to avoid circularization or multimerization of the sRNAs in the pool [46] while the 5′ adapters were chimeric oligonucleotides (5′ atcgtAGGCACCUGAUA 3′ and 5′ atcgtAGGCCACUGAUA 3′; lower case is DNA, upper case is RNA). After each ligation step, the ligated products were selected by size fractionation using denaturing PAGE and purified from the gel as above. The purified-ligated sRNA was reverse-transcribed using SuperScript II reverse transcriptase (Invitrogen). The first-strand cDNA was amplified using Taq DNA polymerase (Perkin Elmer) and 3′ PCR FusionB and 5′ PCR FusionA primers [6]. PCR primers contained the “A” and “B” adaptor sequences used for pyrosequencing. DNA amplicons were gel-purified using 12% native polyacrylamide and eluted as described [46]. Quantity and quality of DNA amplicons were measured using ND-1000 spectrophotometer (Nanodrop) and Experion Automated Electrophoresis System (BioRad), respectively. Same quantity of DNA amplicon from each library was pooled and sequenced by Lifesequencing (http://lifesequencing.com) using 454 GS FLX Technology (454 Life Sciences).

### sRNA analysis: identification of miRNAs and miRNA target prediction

Raw sequences were parsed from FASTA formatted files and assigned to specific libraries. The adapter sequences in the raw reads were removed by using Perl scripts and the bioperl library (http://bioperl.org/). After trimming off the adapters, all sequences were blasted against plant repeat databases to discard abundant non-coding RNAs (rRNA, tRNA, snRNA, and snoRNA) (http://rfam.sanger.ac.uk/ and http://plantrepeats.plantbiology.msu.edu/). Reads of low sequence complexity (less than 3 different bases) and reads outside the 18–25 nt size range were also removed using in-house Perl scripts. The remaining filtered unique sequences were then compared to known mature and precursor miRNAs (pre-miRNAs) from other plant species deposited in miRBase database (http://www.mirbase.org/) using the miRProf tool (UEA sRNA toolkit) (http://srna-tools.cmp.uea.ac.uk/index.php) allowing up to three mismatches and 5′ or 3′ overhanging bases. miRNA-matching cDNA sequences were subjected to stem-loop structure prediction using *mfold* version 3.2 [81]. Predictions were made using RNA sequences containing 50–200 nucleotides on either side of the candidate miRNA. In case no apparent local foldback structure was predicted for a given sequence, larger upstream and downstream sequences were used for mfolding. Criteria for recognition of candidate structured precursors were those suggested by [82].

Target genes of miRNAs were predicted using the online tool psRNATarget (http://bioinfo3.noble.org/psRNATarget/index.php) conforming to parameters previously suggested by [50]. This tool uses an iterative parallel Smith-Waterman algorithm and a weighted scoring schema in which mismatched bases were penalized according to their type and location in the alignment. Mismatches to the 5′ and central regions of the miRNA were preferentially penalized compare to mismatches to the 3′ region of the miRNA. The olive cDNA sequence collection was used to predict the targets (Oleagen web site, http://chirimoyo.ac.uma.es/srs/srs). Functions of the predicted targets were assigned manually based on the function of the best hit from the Blast homology search against the TAIR10 Transcript sequence database.

The raw and processed sequencing data have been deposited into NCBI Gene Expression Omnibus under accession number GSE27093.

### RNA blot assay, PCR and stem-loop quantitative RT-PCR

Blot hybridization of normalized total RNA was performed as described [83]. Oligonucleotides complementary to olive sRNA sequences were end-labeled with [γ-^32^P] ATP using T4 polynucleotide kinase (New England Biolabs). Unincorporated nucleotides were removed using Micro Bio-Spin Chromatography columns (Bio-Rad). Ethidium bromide staining of gels before blot transfer was used to visualize ribosomal RNA and monitor equivalent loading of RNA samples. PCR-based amplification of sRNAs from the amplified sRNA libraries was done as described [42]. An oligonucleotide complementary to the 5′ linker region was used with a 3′oligonucleotide complementary to the particular candidate sRNA.

For quantitative stem-loop RT-PCR, reverse transcription reactions were performed as describe previously with some modifications [84]. Each reaction solution (final volume 20 µl) contained 1 µg of total RNA, 2 mM stem-loop miR159 RT primer (5′ GCCTCTCATGCTGACGAATTTTGAGAGGCTAGAGCTCC 3′, Roche) or miR167 RT primer (5′ GCCTCTCATGCTGACGAATTTTGAGAGGCTAGATCA 3′), 5× Transcriptor buffer, 0.25 mM each of dNTPs, 0.5 U/µl of Transcriptor reverse transcriptase (Roche) and 1 U/µl of RNase out (Roche). RT reactions were incubated in a thermocycler for 30 min at 16°C, 30 min at 42°C and 5 min at 85°C. All reactions, including RT minus controls, were run in duplicate. The RT products were diluted to 200 ng/µl to avoid potential primer interference in the following qPCR reaction. qPCR was performed on a Corbett RG6000 thermocycler in a final volume of 15 µl. The reaction included 3 µl of diluted RT product, 2× FastStart Universal Probe Master Mix (Roche), 0.2 mM TaqMan probe complementary to miR159 (5′ FAM-TTGAGAGGCTAGAGCTCCCTTCA-BBQ 3′, Roche) or to miR167 (5′ FAM-TTGAGAGGCTAGATCATGCTGGC-BBQ 3′, Roche) and 0.5 mM of each PCR primer (miR159-F 5′ GAATTCGACCCTTTGGATTG 3′, miR167-F 5′ ATCAGTAGTGCTTGAAGCTGC 3′ and miR-R 5′ GCCTCTCATGCTGACGAAT 3′, Roche). The reactions were incubated in 0.1 ml tubes (Corbett) at 95°C for 10 min, followed by 40 cycles of 95°C for 15 s and 60°C for 1 min. To minimize sample variability we analyzed two independent biological replicates. All reactions were run in duplicate and in three different PCR runs. For comparison purposes, relative miRNA accumulation was estimated based on the recorded threshold cycle (Ct) that is defined as the fractional cycle number at which the fluorescence signal passes a fixed threshold. The concentration of oeu-miR159 in samples of juvenile and adult shoots from ‘Picual’×‘Arbequina’ was calculated by converting the Ct into an absolute copy number using a standard curve from diluted series of a synthetic ath-miR159a RNA oligo and the RotorGene 6000 software (Corbett). To determine significant differences among samples we applied a One Way ANOVA analysis followed by a Duncan test using Statgraphic Plus 5.1 software.

### miRNA-guided cleavage validation

A modified RLM-RACE was used for mapping internal miRNA-directed cleavage sites on predicted cDNA targets [12]. Total RNA was directly ligated to the 5′ RNA adapter without any further enzymatic pretreatment. Ligated RNA was reverse transcribed using gene specific primers (GSP) that annealed ∼300 nts downstream of the predicted cleavage site within the target mRNA. PCR amplification of the first-strand cDNA was done using a reverse GSP and a forward primer derived from the RNA adapter sequence. The 5′ RACE amplification products were then gel-purified, cloned and sequenced.

## Supporting Information

Figure S1 **Identification of rRNA-derived sRNAs in olive sRNA libraries.** (*A*) Predicted secondary structure of two representative sRNA-containing 18S (oeu-siRNA1) and 26S (oeu-siRNA2) rRNA regions identified in our sequenced set. The sequence of each olive sRNAs is highlighted in bold. (*B*) Expanded diagram of the olive cDNA contig #6083 and stem-loop-like structure. The locations corresponding to the oligonucleotides used as primers for PCR-based detection of sRNAs derived from this locus in the libraries are shown. PCR amplification was done using a 5′ primer for the 5′ adapter sequence used for sRNA library construction and a 3′ primer specific for each sRNA. Note that all but primer #3 rendered sequence-specific amplification products suggestive of broadly generation of sRNAs from the rRNA precursor. PCR control reactions without DNA template (−) are indicated.
(TIF)

Figure S2 **Prediction of secondary structures for known miRNAs precursor in olive.** Hairpin secondary structures for the olive sequence regions around which conserved miRNAs are predicted to be encoded. The putative miRNA sequences identified through deep sequencing of olive sRNAs are highlighted in red bold.
(TIF)

Figure S3 **Prediction of secondary structures of putative novel olive-specific miRNA precursors.** The sRNA sequences identified as potential novel and olive-specific miRNAs are shown in red bold.
(TIF)

Figure S4 **Representation of tasiARF-containing** ***TAS3*** **olive cDNA contigs.** Schematic alignment of olive cDNAs predicted as putative *TAS3* tasiARF precursors. Three different olive loci contain nearly-identical, adjacent tasiARF sequences homologous to Arabidopsis TAS3 5′ D7(+) and TAS3 5′ D8(+). Dual miR390 complementary sites flanking the tasiRNA regions are shown for contigs #48241 and #45914. cDNA sequence of contig #63271 was restricted to the tasiARF region and therefore outside regions containing putative miR390 complementary sites were not represented in the cDNA clone. The regions corresponding to the 5′ miR390 complementary site, tasiARFs, and the 3′ miR390 complementary site are expanded. tasiARFs and miR390 binding sites are indicated. Alignments and color-coded based on the confidence of the local alignment were generated using T-Coffee and its CORE function.
(TIF)

Table S1 **Predicted novel miRNA candidates in** ***O. europaea*** **.**
(DOC)

Table S2 **Predicted olive cDNA targets for candidate olive miRNAs.**
(DOC)
