# Supplementary figures and images for: High-Throughput Sequencing of RNA Silencing-Associated Small RNAs in Olive (Olea europaea L.)
Source: PLoS One. 2011 Nov 28;6(11):e27916. doi: 10.1371/journal.pone.0027916 (PMC3225373; doi:10.1371/journal.pone.0027916)

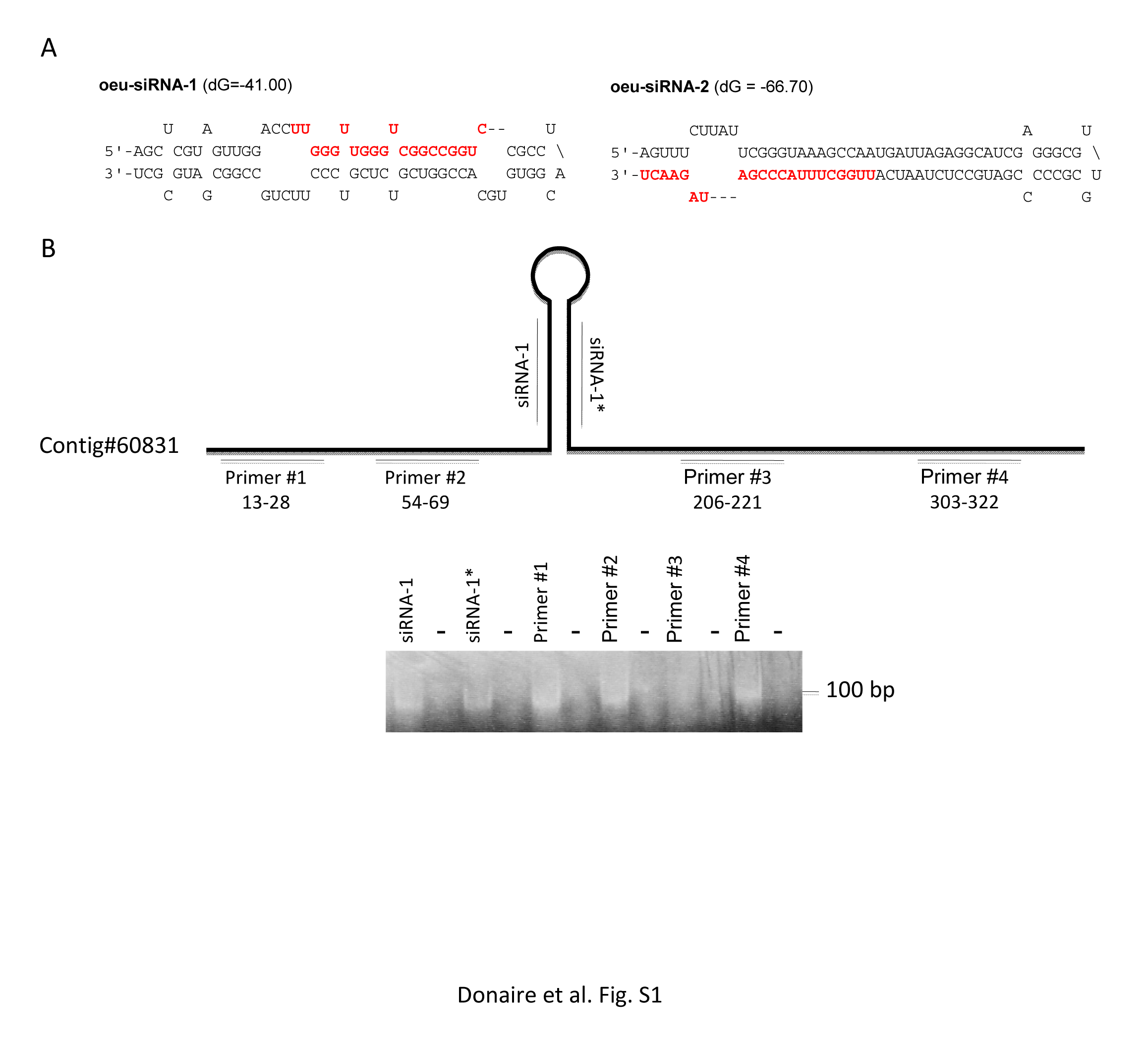

Supplement: Figure S1 — Identification of rRNA-derived sRNAs in olive sRNA libraries. (A) Predicted secondary structure of two representative sRNA-containing 18S (oeu-siRNA1) and 26S (oeu-siRNA2) rRNA regions identified in our sequenced set. The sequence of each olive sRNAs is highlighted in bold. (B) Expanded diagram of the olive cDNA contig #6083 and stem-loop-like structure. The locations corresponding to the oligonucleotides used as primers for PCR-based detection of sRNAs derived from this locus in the libraries are shown. PCR amplification was done using a 5′ primer for the 5′ adapter sequence used for sRNA library construction and a 3′ primer specific for each sRNA. Note that all but primer #3 rendered sequence-specific amplification products suggestive of broadly generation of sRNAs from the rRNA precursor. PCR control reactions without DNA template (−) are indicated. (TIF) [file pone.0027916.s001.tif]

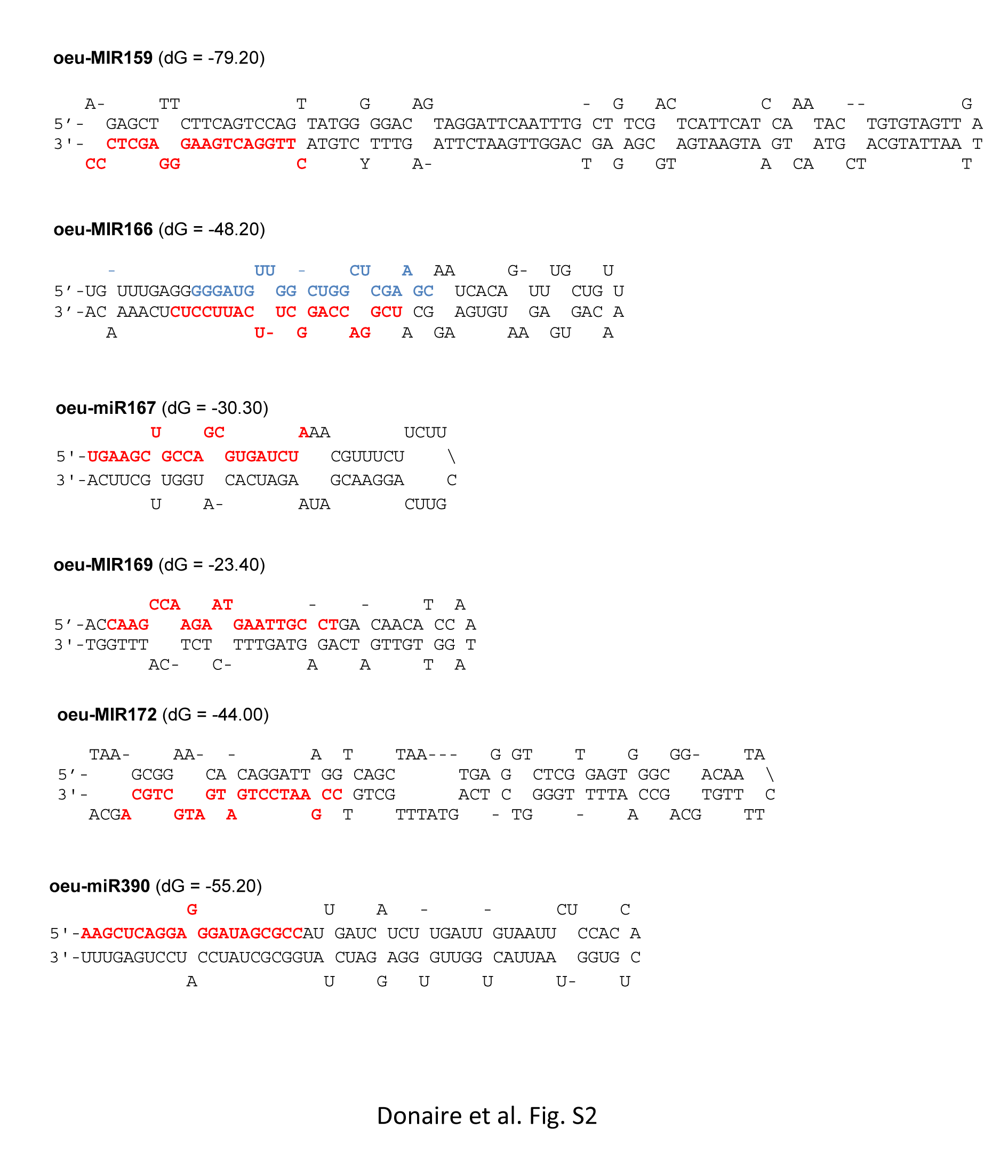

Supplement: Figure S2 — Prediction of secondary structures for known miRNAs precursor in olive. Hairpin secondary structures for the olive sequence regions around which conserved miRNAs are predicted to be encoded. The putative miRNA sequences identified through deep sequencing of olive sRNAs are highlighted in red bold. (TIF) [file pone.0027916.s002.tif]

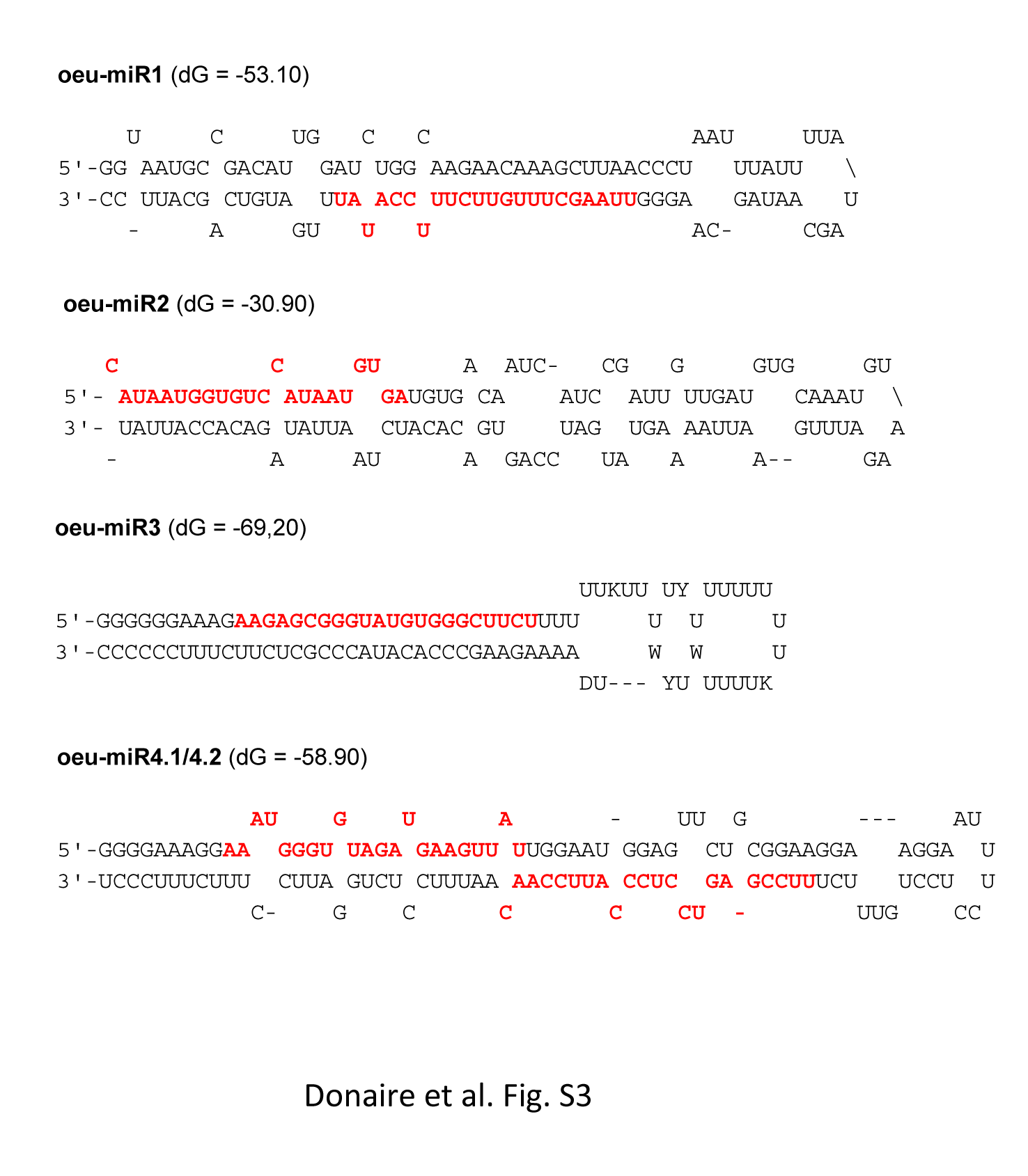

Supplement: Figure S3 — Prediction of secondary structures of putative novel olive-specific miRNA precursors. The sRNA sequences identified as potential novel and olive-specific miRNAs are shown in red bold. (TIF) [file pone.0027916.s003.tif]

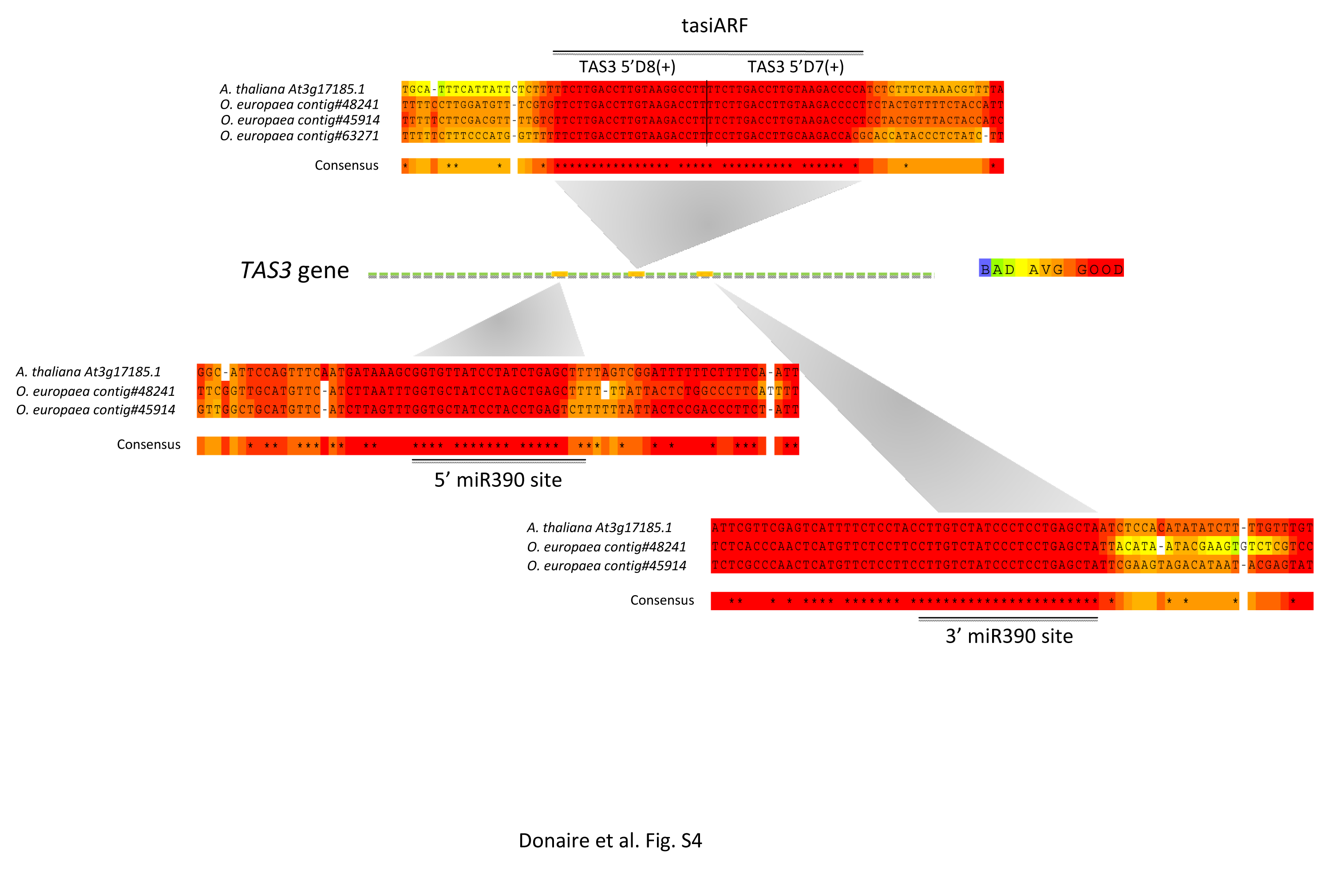

Supplement: Figure S4 — Representation of tasiARF-containing TAS3 olive cDNA contigs. Schematic alignment of olive cDNAs predicted as putative TAS3 tasiARF precursors. Three different olive loci contain nearly-identical, adjacent tasiARF sequences homologous to Arabidopsis TAS3 5′ D7(+) and TAS3 5′ D8(+). Dual miR390 complementary sites flanking the tasiRNA regions are shown for contigs #48241 and #45914. cDNA sequence of contig #63271 was restricted to the tasiARF region and therefore outside regions containing putative miR390 complementary sites were not represented in the cDNA clone. The regions corresponding to the 5′ miR390 complementary site, tasiARFs, and the 3′ miR390 complementary site are expanded. tasiARFs and miR390 binding sites are indicated. Alignments and color-coded based on the confidence of the local alignment were generated using T-Coffee and its CORE function. (TIF) [file pone.0027916.s004.tif]
