# Supplementary material for: High-Throughput Sequencing of RNA Silencing-Associated Small RNAs in Olive (Olea europaea L.)
Source: PLoS One. 2011 Nov 28;6(11):e27916. doi: 10.1371/journal.pone.0027916 (PMC3225373; doi:10.1371/journal.pone.0027916)
Supplement: Table S1 — Predicted novel miRNA candidates in O. europaea . (DOC) [file pone.0027916.s005.doc]

| **Table S1**. Predicted novel miRNA candidates in *O. europaea* | | | | | | | |
| --- | --- | --- | --- | --- | --- | --- | --- |
| miRNA | Sequence (5'-3') | Length (nts) | Juvenile | Adult | Olive cDNA | Precursor length (nts) | dG (Kcal/mol) |
|  |  |  |  |  |  |  |  |
| oeu-miR1 | TTAAGCTTTGTTCTTTCCATAT | 22 | 1 | 0 | Contig #68483 | 107 | -53,10 |
| oeu-miR2 | CATAATGGTGTCCATAATGTGA | 22 | 1 | 0 | Contig #6733 | 111 | -30,90 |
| oeu-miR3 | AAGAGCGGGTATGTGGGCTTCT | 22 | 1 | 0 | Contig #32671 | 97 | -69,20 |
| oeu-miR4.1 | AAATGGGTGTAGATGAAGTTAT | 22 | 1 | 0 | Contig #20467 | 124 | -58,90 |
| oeu-miR4.2 | TTCCGAGTCCTCCCATTCCAAC | 22 | 0 | 1 | Contig #20468 | 124 | -58,90 |
|  |  |  |  |  |  |  |  |

The number of times a sequence was sampled in juvenile and adult shoots is indicated. miRNA-matching olive cDNAs with characteristic fold-back structures are indicated. Expression of oeu-miR4.2 was confirmed by northern hybridization.
