# Supplementary material for: High-Throughput Sequencing of RNA Silencing-Associated Small RNAs in Olive (Olea europaea L.)
Source: PLoS One. 2011 Nov 28;6(11):e27916. doi: 10.1371/journal.pone.0027916 (PMC3225373; doi:10.1371/journal.pone.0027916)
Supplement: Table S2 — Predicted olive cDNA targets for candidate olive miRNAs. (DOC) [file pone.0027916.s006.doc]

| **Table S2**. Predicted olive cDNA targets for candidate olive miRNAs | | | |
| --- | --- | --- | --- |
|  |  |  |  |
| miRNA | Predicted olive cDNAs | Score | Blast TAIR (predicted function) |
|  |  |  |  |
| oeu-miR1 | Contig #85488.6 | 1 | Unknown protein |
|  | Contig #68483.6 | 2 | " |
|  | Contig #40930.6 | 2.5 | " |
|  | Contig #60806.6 | 2.5 | " |
|  | Contig #76993.6 | 2.5 | " |
|  | Contig #43724.6 | 3 | " |
|  | Contig #9427.6 | 3.5 | " |
|  | Contig #29627.6 | 3.5 | " |
|  | Contig #95821.6 | 3.5 | " |
| oeu-miR2 | Contig #87452.6 | 0.5 | " |
|  | Contig #98110.6 | 1 | " |
|  | Contig #11283.6 | 1.5 | " |
|  | Contig #36399.6 | 1.5 | " |
|  | Contig #68499.6 | 1.5 | " |
|  | Contig #89697.6 | 2 | " |
|  | Contig #41650.6 | 2.5 | " |
|  | Contig #53711.6 | 2.5 | " |
|  | Contig #92302.6 | 2.5 | " |
|  | Contig #97841.6 | 2.5 | " |
|  | Contig #80295.6 | 2.5 | " |
|  | Contig #95021.6 | 3 | " |
|  | Contig #97841.6 | 3 | " |
|  | Contig #19295.6 | 3.5 | " |
|  | Contig #19462.6 | 3.5 | " |
|  | Contig #1952.6 | 3.5 | " |
|  | Contig #6856.6 | 3.5 | " |
|  | Contig #93448.6 | 3.5 | " |
| oeu-miR3 | Contig #26273.6 | 2.5 | " |
|  | Contig #32413.6 | 2.5 | " |
|  | Contig #23424.6 | 2.5 | " |
|  | Contig #58522.6 | 3 | " |
|  | Contig #28202.6 | 3.5 | " |
|  | Contig #43275.6 | 3.5 | " |
| oeu-miR4.1 | Contig #19871.6 | 3 | " |
|  | Contig #30618.6 | 3 | " |
|  | Contig #38690.6 | 3 | " |
|  | Contig #85111.6 | 3 | " |
|  | Contig #25356.6 | 3.5 | " |
|  | Contig #47490.6 | 3.5 | " |
|  | Contig #68547.6 | 3.5 | " |
|  | Contig #84476.6 | 3.5 | " |
|  | Contig #98917.6 | 3.5 | " |
|  | Contig #99590.6 | 3.5 | " |
|  | Contig #17958.6 | 3.5 | " |
|  | Contig #28527.6 | 3.5 | " |
| oeu-miR4.2 | Contig #59848.6 | 2 | " |
|  | Contig #67394.6 | 2 | " |
|  | Contig #87529.6 | 2.5 | " |
|  | Contig #26818.6 | 2.5 | " |
|  | Contig #38176.6 | 2.5 | " |
|  | Contig #110703.6 | 3 | " |
|  | Contig #114938.6 | 3 | " |
|  | Contig #115479.6 | 3 | " |
|  | Contig #30739.6 | 3 | " |
|  | Contig #114497.6 | 3.5 | " |
|  | Contig #116369.6 | 3.5 | " |
|  | Contig #126238.6 | 3.5 | " |
|  | Contig #57999.6 | 3.5 | " |
|  | Contig #60962.6 | 3.5 | " |
|  | Contig #64442.6 | 3.5 | " |
|  | Contig #76062.6 | 3.5 | " |
|  | Contig #111984.6 | 3.5 | " |
|  | Contig #113516.6 | 3.5 | " |
|  | Contig #121219.6 | 3.5 | " |
|  | Contig #30692.6 | 3.5 | " |
|  | Contig #42548.6 | 3.5 | " |
|  |  |  |  |

Versions 6 and 7 of the olive cDNA database were used for Blast search and indicated at the end of each contig name. Scores calculated using pSRNATarget (http://bioinfo3.noble.org /psRNATarget/index.php) as suggested by {Allen, 2005 #4}.
